# The fingerprints of climate warming on cereal crops phenology and adaptation options

**DOI:** 10.1038/s41598-020-74740-3

**Published:** 2020-10-22

**Authors:** Zartash Fatima, Mukhtar Ahmed, Mubshar Hussain, Ghulam Abbas, Sami Ul-Allah, Shakeel Ahmad, Niaz Ahmed, Muhammad Arif Ali, Ghulam Sarwar, Ehsan ul Haque, Pakeeza Iqbal, Sajjad Hussain

**Affiliations:** 1 Department of Agronomy, Bahauddin Zakariya University, Multan, 60800 Pakistan; 2 Department of Agricultural Research for Northern Sweden, Swedish University of Agricultural Sciences, 90183 Umeå, Sweden; 3 Department of Agronomy, Pir Mehr Ali Shah, Arid Agriculture University, Rawalpindi, 46300 Pakistan; 4 Agriculture Discipline, College of Science Health, Engineering and Education, Murdoch University, 90 South Street, Murdoch, WA 6150 Australia; 5 College of Agriculture, Bahauddin Zakariya University, Bahadur Sub-campus, Layyah, 31200 Pakistan; 6 Department of Soil Science, Bahauddin Zakariya University, Multan, 60800 Pakistan; 7 Cotton Botanist, Cotton Research Station, Ayub Agricultural Research Institute, Faisalabad, 38000 Pakistan; 8 Citrus Research Institute Sargodha, Sargodha, 40100 Pakistan; 9 Department of Botany, University of Agriculture Faisalabad, Faisalabad, Pakistan; 10 Department of Horticulture, Bahauddin Zakariya University, Multan, Pakistan

**Keywords:** Agroecology, Climate-change impacts

## Abstract

Growth and development of cereal crops are linked to weather, day length and growing degree-days (GDDs) which make them responsive to the specific environments in specific seasons. Global temperature is rising due to human activities such as burning of fossil fuels and clearance of woodlands for building construction. The rise in temperature disrupts crop growth and development. Disturbance mainly causes a shift in phenological development of crops and affects their economic yield. Scientists and farmers adapt to these phenological shifts, in part, by changing sowing time and cultivar shifts which may increase or decrease crop growth duration. Nonetheless, climate warming is a global phenomenon and cannot be avoided. In this scenario, food security can be ensured by improving cereal production through agronomic management, breeding of climate-adapted genotypes and increasing genetic biodiversity. In this review, climate warming, its impact and consequences are discussed with reference to their influences on phenological shifts. Furthermore, how different cereal crops adapt to climate warming by regulating their phenological development is elaborated. Based on the above mentioned discussion, different management strategies to cope with climate warming are suggested.

## Introduction

**Figure 1:**
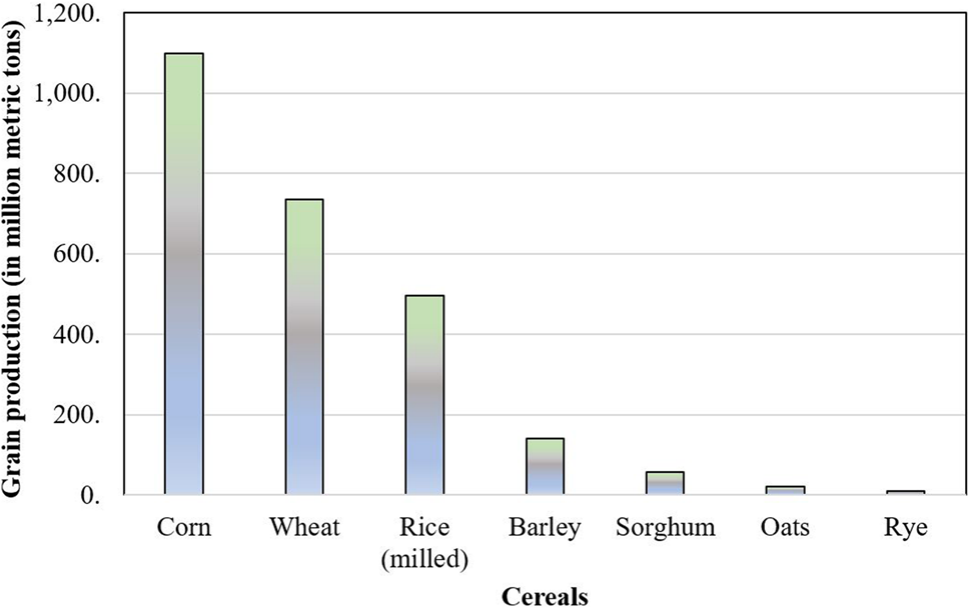
Cereals grain production.

Cereal crops are typically grasses grown for their edible grains. Globally, cereal grains are produced in higher quantities than any other type of crop and deliver more food energy to human beings and livestock than other crops^1,2^ (Fig. 1). The cereal production of the top 20 countries in the world is presented in Table 1.

**Table 1:** World cereal production of top 20 countries during 2012–2016 (metric tonnes). (*Source*: FAOSTAT).

Sr. No	Countries & Years	2016	2015	2014	2013	2012
1	China	580,898,372	572,045,000	557,417,296	552,691,792	539,346,800
2	USA	475,983,881	431,865,458	442,849,090	434,308,450	356,210,124
3	India	294,711,871	284,333,000	296,010,000	294,909,510	293,290,000
4	Russia	117,749,733	102,450,556	103,141,153	90,369,572	68,757,301
5	Indonesia	97,667,060	95,010,276	89,854,891	89,791,565	88,443,150
6	Brazil	84,128,482	106,029,517	101,402,184	100,901,726	89,908,244
7	Argentina	67,024,441	55,650,667	55,876,126	50,703,913	47,449,657
8	Ukraine	65,211,480	59,623,480	63,378,190	62,679,750	45,742,860
9	Bangladesh	56,388,892	54,904,305	55,759,375	54,344,578	52,798,736
10	Canada	55,251,252	53,361,100	51,535,801	66,405,701	51,799,100
11	France	54,654,565	72,875,854	72,579,315	67,537,681	68,341,731
12	Vietnam	48,685,089	50,393,869	50,178,378	49,231,389	48,712,795
13	Germany	45,364,400	48,866,800	52,010,400	47,757,100	45,396,500
14	Pakistan	43,075,694	41,081,682	41,895,811	40,109,711	36,496,350
15	Mexico	38,466,082	34,704,514	36,526,602	33,210,301	33,614,212
16	Turkey	35,276,615	38,632,438	32,708,005	37,475,610	33,370,865
17	Australia	35,230,376	37,195,488	38,419,610	35,587,287	43,362,415
18	Thailand	30,420,758	32,786,514	37,751,337	41,962,227	43,367,009
19	Poland	29,849,223	28,002,726	31,945,433	28,455,154	28,543,870
20	Myanmar	28,109,023	28,634,939	28,792,954	28,626,124	28,344,271

Wheat (*Triticum aestivum* L.), rice (*Oryza sativa* L.), maize (*Zea mays* L.), barley (*Hordeum vulgare* L.) and oat (*Avena sativa* L.) are important cereal crops in most countries. Other includes rye (*Secale cereal*), sorghum (*Sorghum bicolor*), and millet (*Pennisetum glaucum)*. These crops belong to the family Poaceae or Gramineae subfamily Oryzeae (Genus Oryza: Rice), Triticeae (Genus Triticum: Wheat, Hordeum: Barley, Scecale: Rye), Aveneae (Genus Avena: Oat), Paniceae (Genus Pennisetum: Millet), Andropgonee (Genus Sorghum, Zea: Maize, Coix: Jobs’s tears) and Cynodonteae (Genus Eleusine, Ragi). They contribute over 50% of world's total production of cereals in million tonnes^3–6^. Cereals as whole grain are a rich source of minerals, carbohydrates, vitamins, oils, fats, protein, fiber content and are preferred for consumption^7–10^ (Table 2).

**Table 2:** Nutritional values of cereals per 100 g. (*Source*: Price and Welch^9^).

Nutritional values/Crops	Rice	Maize	Wheat	Barley	Oat	Rye	Sorghum	Millet
Water (g)	13.9	12.2	14	10.6	8.5	15	12	11
Energy (kJ)	1518	1517	1320	1535	1644	1268	1422	1468
Energy (kcal)	357	357	310	360	388	298	335	346
Carbohydarte (g)	81.3	77.2	63.9	83.6	69.4	65.9	69.9	68.1
Protein (g)	6.7	9.4	12.7	7.9	11.8	8.2	10.7	11.8
Fat (g)	2.8	3.3	2.2	1.7	9	2	3.3	4.8
Dietary fiber (g)	1.9	2.2	9	5.9	7	11.7	7.5	6.9
**Essential amino acid (g/100 g protein)**								
Phenylalanine	5.2	4.8	4.6	5.2	5.4	5	5.1	5.5
Histidine	2.5	2.9	2	2.1	2.4	2.4	2.1	2
Isoleucine	4.1	3.6	3	3.6	4.2	3.7	4.1	3.8
Leucine	8.6	12.4	6.3	6.6	7.5	6.4	14.2	10.9
Lysine	4.1	2.7	2.3	3.5	4.2	3.5	2.1	2.7
Methionine	2.4	1.9	1.2	2.2	2.3	1.6	1	2.5
Threonine	1	3.9	2.4	3.2	3.3	3.1	3.3	3.7
Tryptophan	1.4	0.5	2.4	1.5	–	0.8	1	1.3
Valine	5.8	4.9	3.6	5	5.8	4.9	5.4	5.5
**Fatty acid profiles (g/100 g food)**								
Total fat	2.8	–	1.2	1.7	9.2	2	–	–
Saturated fatty acids	0.74	–	0.16	0.29	1.61	0.27	–	–
Cis-monounsaturated fatty acids	0.66	–	0.13	0.14	3.34	0.21	–	–
Polyunsaturated fatty acids:								
Total cis	0.98	–	0.51	0.77	3.71	0.95	–	–
n−6 (as 18:2)	0.94	–	0.48	0.7	3.52	0.82	–	–
n−3	0.04	–	0.03	0.07	0.19	0.13	–	–

Nutrient	White	Brown	Wholemeal					
**Typical Nutrient composition per 100 g of cereal bread**								
Energy (kcal/kJ)	219/931	207/882	217/922					
Protein (g)	7.9	7.9	9.4					
Carbohydrate (g)	46.1	42.1	42					
Total sugars (g)	3.4	3.4	2.8					
Starch (g)	42.7	38.7	39.3					
Fat (g)	1.6	2	2.5					
Fibre (g)	1.9	3.5	5					
Thiamine (mg)	0.24	0.22	0.25					
Niacin equivalents (mg)	3.6	4.9	6.1					
Folate (μg)	25	45	40					
Iron (mg)	1.6	2.2	2.4					
Calcium (mg)	177	186	106					

Local and regional climatic conditions are a primary determinant of agronomic crop productivity. Plant metabolic processes are controlled by weather variables like maximum and minimum temperature, solar radiation, carbon dioxide concentration and availability of water^11–17^. Cereal crop production is influenced by extreme climatic conditions, like heat waves, storms, drought, salinity and flooding^17–22^. Similarly, Nicholson et al.^23^ reported change in the seasonality of rainfall in large part of Africa. Similar kind of spatiotemporal changes in aridity and shifts of dryland in Iran was concluded by Pour et al.^24^ which could have severe effect on agricultural production and food security. The release of greenhouse gases due to different anthropogenic activities have affected agricultural activities across geographical regions^7^. Distant changes in air temperature resulted to significant change in plant phenology^8,25–32^. During the previous century, but predominantly over the most recent decades, the earth has experienced noteworthy climate change, particularly warming trends across globe. The average air temperature has increased almost 0.95 °C from 1980 to 2018, and it is predicted to increase almost 3.0–5.0 °C (depending on region) by the end of this century. In the meantime, world population has grown considerably and will continue to grow at an increasing rate. It is expected that the world will need 70% more food by the middle of the current century^15,33^.

Food security and climate change mitigation are major challenges in developing countries. Changes in management practices such as fertilizer use efficiency, use of organic fertilizers, use of legume with grasses, optimization of irrigation water, plant breeding and genetic modifications and selection of cultivars in field could be good mitigation strategies to coup climate change^15,34,35^. These adaptive mitigation strategies could help to design resilience systems to future climate change. Similarly, promotion of climate smart agriculture (CSA) could help to mitigate the challenges of climate change. CSA aims to transform and redirect agricultural system to support sustainable development and food security under changing climate. It integrates three elements of sustainable development (economic, social, and environmental) by addressing food security and climate change. It has three main pillars (i) Sustainably increasing agricultural productivity and incomes (ii) Adapting and building resilience to climate change and (iii) Reducing and/or removing greenhouse gas emissions. Climate change can greatly influence overall food security by influencing cereal crop phenology and changing spatial allocation of crops. According to earlier work, it has been predicted that a 2.0 °C rise in average temperature can lead to a more than 20 to 40% reduction in cereal grain production, particularly in Asia and Africa. Furthermore, temperature rise can have either negative or positive impacts on crop productivity depending upon regions^36–48^. Shift in the seasonality is the one of the big example of rise in temperature^49^.

Temperature is the most important environmental attribute influencing growth and development, and hence, ultimate productivity of agronomic cereal crops. Rise in temperature also leads to higher evapotranspiration, more crop water and nutrients loss thus resulting to lower water use and nitrogen use efficiency. The effect of temperature on crop phenology could be documented by using GDD or heat units. Heat units, expressed in growing degree-days (GDD), are frequently used to describe the timing of biological processes. The basic equation used is

$$GDD= \left[\frac{\left({T}_{maximum}-{T}_{minimum}\right)}{2}\right]-{T}_{base}$$

where *T*_maximum_ and *T*_minimum_ are daily maximum and minimum air temperature, respectively, and *T*_base_ is the base temperature. It also controls the rate of development from emergence to physiological maturity^50–54^. All cereal crops have a basic requirement of temperature for completion of a given phenological phase, or the entire life cycle^55–58^. The daily maximum and minimum temperature play a significant role in determining the optimum sowing time or window and defining the seasonal duration, thus both can affect the achievable yield, produce quality, and along with their sustained productivity^59–64^. Normally, the longer the optimum growing season, then higher the duration for maximum grain production. Luo^65^, reviewed threshold temperature of different crops and stated that identification of threshold temperature is very important aspect of climate change risk assessment. Furthermore, cardinal T (Base T = T_base_, optimum T = T_opt1_ and T_opt2_, and failure point T = T_fp_) and lethal T (Lethal minimum T = T_lmin_ and lethal maximum T = T_lmax_) have strong association with crop production. Cardinal and extreme temperature thresholds for the major cereal crops were reviewed by Ramirez-Villegas et al*.*^66^ as shown in Table 3. Threshold temperature for the different phenological stage/phase of the wheat crop have been presented in Table 4.

**Table 3:** Cardinal (Base (*T*_b_), optimum (*T*_opt_) and maximum (*T*_max_)) and extreme temperature thresholds (Ceiling vegetative temperature (T_C1_) and Ceiling reproductive temperature (T_C2_)) for cereal crops. (*Source*: Ramirez-Villegas et al^66^).

Crops	T_b_(°C)	T_opt_ (°C)	T_max_ (°C)	T_C1_ (°C)	T_C2_ (°C)
Barley	0.0–5.0	25.0–31.0	50.0	30.0	40.0
Maize	8.0	30.0	38.0	33.0	44.0
Millet	10.0	34.0	40.0	30.0	40.0
Oat	0.0–5.0	25.0–31.0	31.0–37.0	–	–
Rice	20.0	28.0	35.0	22.0	30.0
Rye	0.0–5.0	25.0–31.0	31.0– 37.0	–	–
Sorghum	8.0	34.0	40.0	32.0	44.0
Wheat	0.0	13.2	35.0	34.0	40.0

**Table 4:** Temperatures (Base (*T*_b_), optimum (*T*_opt_) and maximum (*T*_max_)) for different phenological phases and stages in wheat. (*Source*: Porter and Gawith^53^).

Phenological stage/phase	*T*_*b*_ (°C)	*T*_opt_ (°C)	*T*_max_ (°C)
Leaf initiation	− 1.0	22.0	24.0
Shoot growth	*3.0*	*20.3*	> 20.9
Root growth	*2.0*	< *16.3*	> *25.0*
Sowing	5.0	20.0	30.0
Germination/emergence	0–4.5	24.0–28.0	35.0
Vernalization	− 5.0	0.0–12.0	> 12.0
Tillering	< 3.0	6.0–9.0	> 9.0
Double ridges	4.0	20.0	–
Spikelet Initiation	0.0	15.0	20.0–25.0
Terminal spikelet	3.0	8.0–12.0	–
Shoot elongation	< 12.0	15.0–22.0	> 40.0
Heading	3.9	24.3	–
Anthesis	< 10.0	18.0–24.0	> 32.0
Pollination	> 10.0	18.0–24.0	32.0
Grain–filling	12.0	20.0	35.0
Maturity	< 15.0	22.0–25.0	> 32.0

	T_lmin_	T_lmax_	
Lethal Limits	–17.2	47.5	

The predicted changes in temperature during the next 40 to 70 years are expected to be in the range of 2–3 °C in different regions. Intensity and duration of warming trends and heat wave events are predicted to become more extreme in future than at present and during last decade^67–69^. Extreme temperature events have short-term spells of a few days with temperature increases over 5.0 °C above normal. Frost caused sterility and abortion of grain whilst extreme heat caused a decrease in the number of grains and reduced grain filling duration^70–72^. Day-to-day minimum temperatures will rise more rapidly compared to daily maximum temperatures leading to increased mean temperatures. These variations have detrimental effects on yield^52,73^. The work by Srivastava et al.^74^ concluded that maximum temperature affects the maize grain yield more than the minimum temperature under variable CO_2_ levels in both irrigated and rainfed conditions. Furthermore, their study confirmed that maize yield was low for RCP 2.6, 4.5, 6.0, and 8.5 under irrigated as compared to rainfed condition. If such changes in temperature will remain occurring in future, then adaptation strategies such as change in sowing date and cultivars shifts are needed to offset such impacts^75–78^.

Crop phenology is the most significant feature of crop adaptation, and spatial and temporal changes in phenological stages and phases provide well-built indications of the biological influence of thermal trend. Change in phenological seasonality is having both direct and indirect effects on natural vegetation and cereal crop productivity^25,26,79–81^. Measured climate-warming trends have been implicated as a reason for accelerated cereal crop growth rates and reduced growing durations. Since phenology is highly sensitive to climate change and has significant impact on carbon balance. Thus the asymmetric and opposing response of phenology to daytime and night-time was studied by Wang et al.^82^ and concluded that *T*_max_ and night-time *T*_min_ had opposite effects on the timing of start of the season, delayed end of the season and prolonged length of growing season. Furthermore, several earlier studies documented that the intensity of the response of phenological stages and phases of agricultural crops to warming trend is variable at spatio-temporal scales^79,83,84^. Phenology is well known climate change indicator. However, there is a lack of critical analysis of climatic warming impacts on cereal phenology shift and management of climatic impacts to ensure future global food security. Therefore, a major objective of this review is to analyze the phenological fingerprinting of cereal crops in response to climate warming, and to discuss important management strategies to overcome the climate warming impacts on phenological shift of cereal crops.

## Climate warming

**Figure 2:**
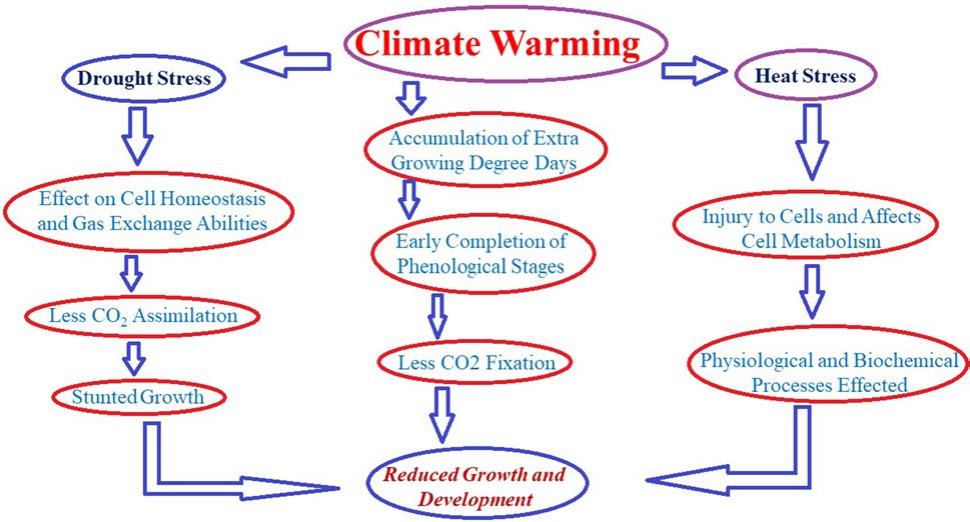
A simple presentation of climate warming effects on growth and development of crop plants.

The global environment is changing with increasing temperature and carbon dioxide concentration [CO_2_]. Generally, burning of fossil fuels are the principle source of climate warming. Jackson et al.^85^ reported that global CO_2_ emissions by fossil fuel burning in 2017 were 36.2 billion tonnes, which reached to 37.1 billion tonnes in 2018^86^. Additionally, CO_2_ emissions due to biomass combustion were approximately 1/3rd of the fossil fuel emissions during 1997–2010^87^. These CO_2_ emissions contribute to global warming and the current fossil fuel reserves have the potential to increase the global temperature up to 2 °C by 2050. Phenology of crop is codetermined by environmental conditions (thermal time requirement) and agronomic practices (Sowing date and cultivar features). Detection of climate warming impact on phenology is not easy mainly because of change in sowing date and introduction of new cultivars. Warming accelerate crop development but it could result to delayed development for long duration cultivar or late sowing date. The impact of climate warming can be mitigated by use of new cultivars with longer thermal time requirement. It has been documented earlier that 1/3rd of the increased temperature impact on phenology was compensated by the introduction of new cultivars with altered temperature requirements. Globally, the climate warming and other stresses are contributing towards yield losses and advocating the necessity of changing the crop calendar (Fig. 2)^57,88–91^. This necessity for changing the crop calendar is mainly because of changes in the timing of plant phenological stages or events that respond to temperature (Fig. 2). This change in cropping calendar might have positive or negative impacts on productivity, depending on type of crop and geographical locations^92^. He et al.^37^ reported delayed sowing dates and use of longer duration cultivars are possible management adaptation options needs to be considered to adapt to climate change. Their results indicated that farmers have a wider sowing window in spring and can select frost tolerance and long growing season cultivars that can mitigate detrimental effects of climate warming. Thus, it is very significant to study the climate warming effect on cereal crops phenology.

## Impact of climate warming on phenological shift

Phenological stages of the crop plant are set very strictly to the seasonality of the environment and are therefore influenced by changes in the environment. Furthermore, the duration of phenological stages is also associated with CO_2_ assimilation, so a shift in phenology may affect crop yield. Tao et al.^38^ explored the climate-crop-region relation for major crops, i.e., rice, maize and wheat over a period of 1951–2002 and reported a trend of 3.2 × 10^5^, − 1.2 × 10^5^, and − 21.2 × 10^5^ t decade^−1^ change in production of rice, maize and wheat respectively. They found different impacts of climate warming in different regions. For instance, in northeast China; rice production increased, and in northeast and north regions maize and wheat yield decreased in all studied regions. Wang et al.^7^ reported shortening of vegetative and reproductive growth phases of double rice grown across southern China in response to temperature increase.

Blecharczyk et al.^93^ investigated phenological and climate warming data of winter rye grown in Poland from 1957 to 2012 and reported an increase of 2 °C in average temperature. During this period, they observed significant delay in sowing dates and an advancement of 4 days decade^−1^ in flowering initiation. These changes occurred due to the relative thermal requirement of the crops and cultivar effects. They also reported compensatory effects of cultivars and management practices on crop phenology in response to climate warming. Increased temperature speeds growth, which leads to reduced yield. Ahmad et al.^94^, Huang and Ji^95^ and Wang et al.^96^ reported negative correlations of temperature rise with all the phenological stages besides cotton yield in central and lower Punjab regions of Pakistan. They further reported that about 30% of the negative consequences of climate warming are compensated by sowing new cotton cultivars with high thermal requirements.

Although climate warming is a long and continuous process, it has accelerated during the industrial revolution i.e., after the mid-twentieth century. With rapid industrialization, emission of CO_2_ increased in the atmosphere and increasing population lead to deforestation to clear new residential area. Earlier crop flowering and maturity have been observed during the last few decades, but crop management also has impacted the effect of climate warming. Siebert and Ewert^52^ reported spatial differences in climatic effects in different regions of Germany due to temperature and day length effects. Compensation of climatic warming effects on cotton, wheat and rice (30, 21 and 35%, respectively) grown in Punjab, Pakistan, using new cultivars with higher thermal requirements are also reported^88,94^. However, they reported spatio-temporal differences in the crop species for response to climate warming. Sorghum (*Sorghum bicolor* L.) is a very important crop in arid, marginal and warm regions throughout the world, and it is affected by climate warming. Srivastava et al.^97^ analyzed the impact of climate change on sorghum using the InfoCrop-SORGHUM simulation model. They reported regional variation in response to climate warming and projected a 7% yield reduction up to 2020. Sultan et al.^98^ assessed near-term climate warming impacts on sorghum yield using different crop models in West Africa. Based on 1961–1990 data, they projected an increase of 2.8 °C in mean temperature of 2031–2060 and robust change in rainfall pattern and projected 16–20% yield reduction in response to the climate changes. Shew et al.^99^ studied climate-warming impact on 71 cultivars across 17 locations in South Africa from 1998 to 2014 through extensive regression models. The outcomes showed that heat drives wheat yield losses. The reduction was 12.5% with an additional one-day (24 h) exposure to temperature above 30 °C. Furthermore, 8.5%, 18.4% and 28.5% yield reduction was observed for uniform warming scenario of + 1 °C, + 2 and + 3 °C respectively. They suggested that warming impact could be reduced by sharing gene pools with wheat breeding programs. Sonkar et al.^100^ reported 7% decrease in wheat yield per 1 °C rise in the mean temperature. Thus, there is a dire need of further research especially on the effects of climate change on the optimum temperatures required for different crop development stages. In short, climate warming has mostly negative impacts on crop species by disturbing their phenology, but these impacts can be compensated or converted into positive effects by proper management, ideotype designing and breeding of fast-growing crops with higher thermal requirements^37,88,101–106^.

## Role of phenological stages and phases in cereal crop grain production

Cereal crop phenology is very sensitive to climate change^88^ as compared to other agronomic crops; consequently, phenological changes are frequently used to measure how climate warming influences the agricultural ecosystems in a particular region^107–109^. Numerous methods have been utilized to elucidate the intensity of the effect of climate change on agronomic cereal crops. The methods include analysis of satellite images on vegetation greenness, measurement of net primary production with Normalized Difference Vegetation Index (NDVI), and particularly, determination of temporal and spatial variations in phenological seasonality^26,79,110–115^. The data on stages and phases of cereal crop phenology and climate meteorological data are collected from phenological networks or through designated observation stations.

### Wheat phenology trend

**Figure 3:**
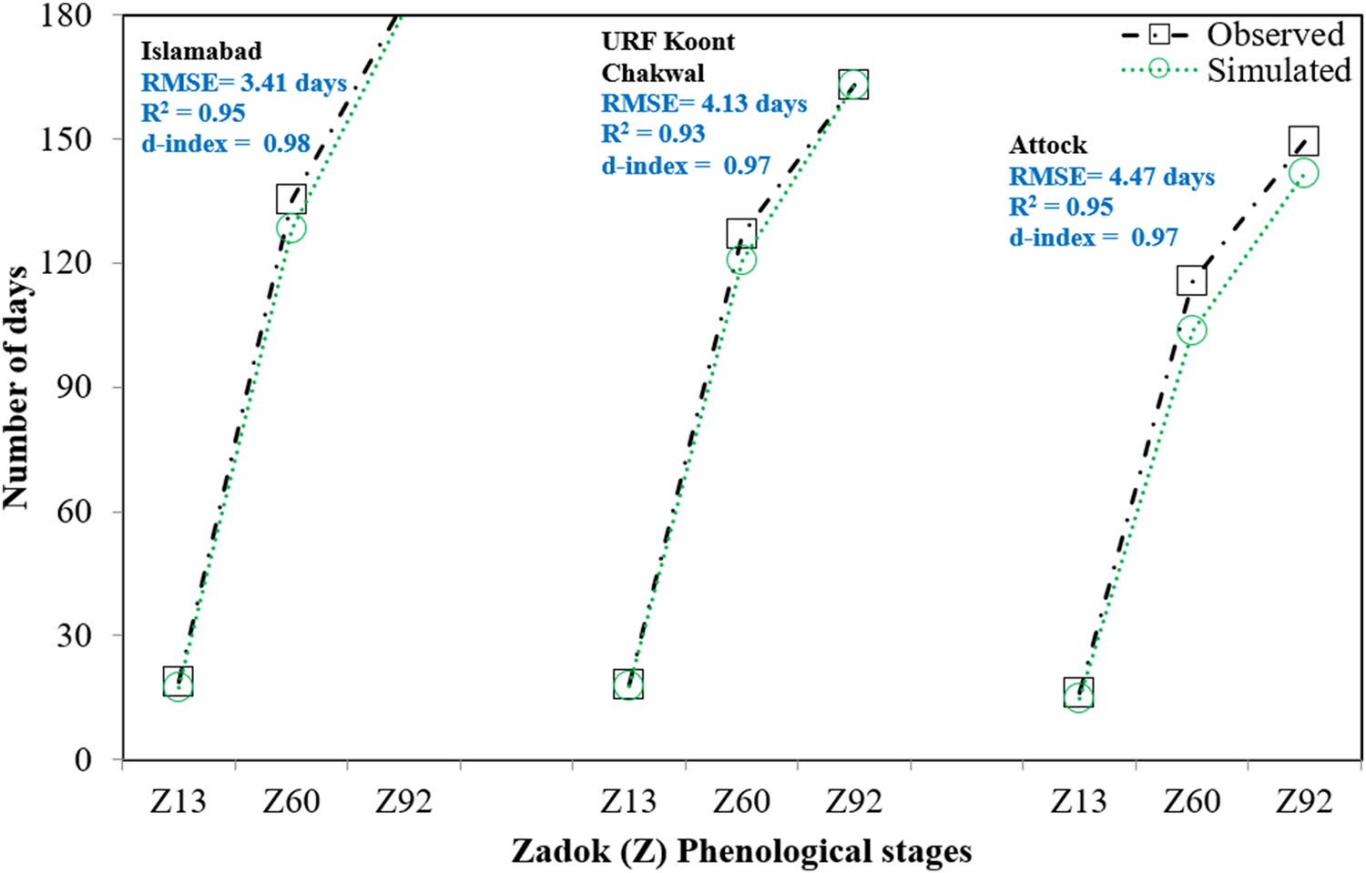
Trend of phenological stages of wheat at three variable climatic sites of rainfed Pothwar.

Wheat phenology has been affected by climate warming worldwide. Identifying phenological responses to climate warming is tough due to regularly shifting sowing dates and the introduction of new cultivars. Wheat phenological trend from sowing to maturity is presented in Table 5. The results showed that it remained maximum in northwest China during 1983 to 2004 (sowing = 13.2 days decade^−1^, emergence = 9.8 days decade^−1^, anthesis = 11.0 days decade^−1^ and maturity = 10.8 days decade^−1^). However, highest trend in delaying of phenological stages was observed in north south regions of China during 1981–2010^116^. The highest reduction in phenological phases was also observed in northeast China^11^. Xiao et al.^117^ observed that since 1981 to 2009, climate-warming in North China Plain caused dates of green-up after winter dormancy, anthesis, and winter wheat maturity to happen in an average of 1.1, 2.7, and 1.4 days earlier decade^−1^, respectively. Wheat phenology was recorded at three variable sites of rainfed Pothwar and it depicted significant variability. The highest days to flowering and maturity was observed at Islamabad while lowest were at Attock which might be due to variability in climatic conditions during wheat growing cycle (Fig. 3). Ahmad et al.^88^ reported that sowing (S), emergence (E), anthesis (A) and maturity (M) for wheat crop were delayed by 9.5, 1.3, 5.3 and 5.4 days deacade^−1^ while phenological-phases S-A, A-M along with S-M were reduced by 5.4, 5.5, 4.6 and 5.7 days decade^−1^ in Pakistan during 1980 to 2014 (Fig. 3). The S-M phase was reduced by 7.2, 10.7, 5.4, 4.0 and 2.7 days decade^−1^ in Spain, Australia, Argentina, Romania and Germany, respectively. In India, Russia and USA, crop phenology was also affected due to climate change^118,119^.

**Table 5:** Cereal crops observed phenology trends across regions (*E* Early, *D* Delay, *S* Sowing, *T* Transplanting, *A* Anthesis, *M* Maturity).

Crop	Country	Period	Phenological stages (early/delay days/decade)				Phenological phases (reduction days/decade)			References
			Sowing	Emergence	Anthesis	Maturity	S-A	A-M	S-M	
Wheat	China	1983–2004	13.2 (E)	9.8 (E)	11.0 (E)	10.8 (E)	16.1	8.2	12.3	^7^
	Pakistan	1980–2014	9.5 (D)	1.3 (D)	5.3 (E)	5.4 (E)	5.5	4.6	5.7	^88^
	China	1981–2005	7.6 (E)	6.3 (E)	2.0 (E)	4.8 (E)	3.8	4.1	5.8	^14^
	Spain	1986–2012	3.8 (E)	2.6 (E)	5.2 (E)	2.9 (E)	4.6	5.1	7.2	^81^
	Australia	1995–2016	3.9 (E)	2.8 (E)	7.5 (E)	5.8 (E)	6.6	7.9	10.7	^126^
	China	1981–2009	1.2 (D)	1.3 (D)	3.7 (D)	3.1 (E)	5.0	3.1	4.3	^127^
	Argentina	1971–2000	3.0 (D)	2.9 (D)	4.2 (D)	4.9 (D)	7.5	6.9	5.4	^25^
	China	1980–2009	4.1 (D)	3.7 (D)	6.3 (D)	8.1 (D)	6.1	2.3	3.6	^128^
	Romania	1971–2006	3.5 (D)	2.5 (D)	2.2 (D)	3.0 (D)	2.3	3.2	4.0	^61^
	China	1981–2010	9.0 (D)	8.5 (D)	11.0 (D)	16.2 (D)	3.7	2.5	1.3	^27^
	China	1981–2009	1.5 (D)	1.7 (D)	2.1 (D)	2.5 (D)	2.0	1.8	3.1	^11,28,42,50,128–133^
	China	1981–2000	3.4 (E)	2.9 (E)	3.0 (E)	3.3 (E)	0.4	0.3	1.0	^38^
	Germany	1952–2013	2.0 (E)	1.8 (E)	4.1 (E)	5.0 (E)	1.9	0.8	2.7	^134,135^
Rice	Pakistan	1980–2014	7.9 (E)	6.6 (E)	5.0 (E)	5.0 (E)	1.4	4.1	6.4	^88^
	Madagascar	2008–2010	5.4 (E)	3.2 (E)	6.2 (E)	4.8 (E)	4.1	3.2	6.2	^108^
	China	1981–2006	1.0 (D)	1.4 (D)	2.7 (D)	3.1 (D)	3.3	1.2	4.1	^70^
	China	1981–2000	5.7 (E)	5.2 (E)	6.2 (E)	3.6 (E)	0.5	2.6	3.1	^38^
	China	1992–2013	2.2 (D)	1.9 (D)	2.8 (D)	3.4 (D)	0.8	1.7	2.4	^136^
	China	1981–2012	4.9 (D)	4.2 (D)	3.8 (D)	5.2 (D)	2.4	3.2	5.1	^137^
	China	1981–2009	3.7 (D)	3.0 (D)	2.0 (D)	4.0 (D)	2.8	1.9	2.2	^138^
	China	1981–2009	6.5 (D)	5.8 (D)	1.5 (D)	2.4 (D)	2.9	1.6	5.2	^139^
Maize	Pakistan	1980–2014	3.0 (D)	1.9 (D)	2.8 (D)	4.4 (D)	5.5	2.2	7.8	^109^
	Pakistan	1980–2014	4.6 (E)	3.7 (E)	7.1 (E)	9.2 (E)	2.4	1.9	4.6	^109,140,141^
	China	1981–2010	5.4 (D)	4.8 (D)	5.2 (D)	7.1 (D)	1.3	0.8	2.2	^142^
	China	1990–2012	10.0 (D)	9.4 (D)	10.5 (D)	5.6 (D)	4.1	3.9	5.7	^143^
	China	1981–2008	8.1 (D)	7.8 (D)	6.2 (D)	3.7 (D)	5.2	2.6	3.7	^136^
	USA	1981–2005	3.9 (E)	3.2 (E)	1.7 (E)	2.9 (E)	2.9	1.8	3.0	^144^
	Germany	1961–2000	4.5 (E)	4.1 (E)	5.6 (E)	8.8 (E)	3.1	7.2	4.9	^8^
	China	1981–2000	1.7 (D)	1.5 (D)	3.3 (D)	5.5 (D)	2.4	2.2	2.8	^38^
	China	1981–2009	5.0 (D)	3.1 (D)	4.0 (D)	6.7 (D)	1.0	2.7	3.5	^27,102^
	China	1992–2013	3.5 (D)	3.2 (D)	1.8 (D)	1.5 (D)	1.9	3.3	1.5	^145,146^
	China	1981–2010	8.7 (D)	6.9 (D)	4.6 (D)	2.2 (D)	4.6	2.4	6.2	^146,147^
	China	1992–2013	1.3 (E)	1.0 (E)	4.1 (E)	2.7 (E)	1.1	2.0	3.0	^145^
Oat	Germany	1959–2009	1.1 (E)	1.8 (E)	9.7 (E)	10.7 (E)	7.9	13.9	9.4	^52^
	Germany	1951–2004	1.5 (E)	1.2 (E)	4.9 (E)	6.4 (E)	3.4	1.5	4.9	^110^
Barley	Lithuania	1961–2015	1.7 (E)	2.8 (E)	1.1 (E)	0.4 (E)	1.0	1.6	2.2	^43^
	Spain	1986–2008	2.8 (D)	1.9 (D)	2.7 (D)	3.5 (D)	1.6	2.1	3.7	^41^
Rye	Poland	1957–2012	2.2 (D)	1.9 (D)	4.0 (D)	3.6 (D)	1.8	1.4	3.2	^125^
	Germany	1960–2013	1.0 (E)	1.2 (E)	1.8 (E)	1.6 (E)	2.9	3.1	4.5	^60^

### Rice phenology trend

**Figure 4:**
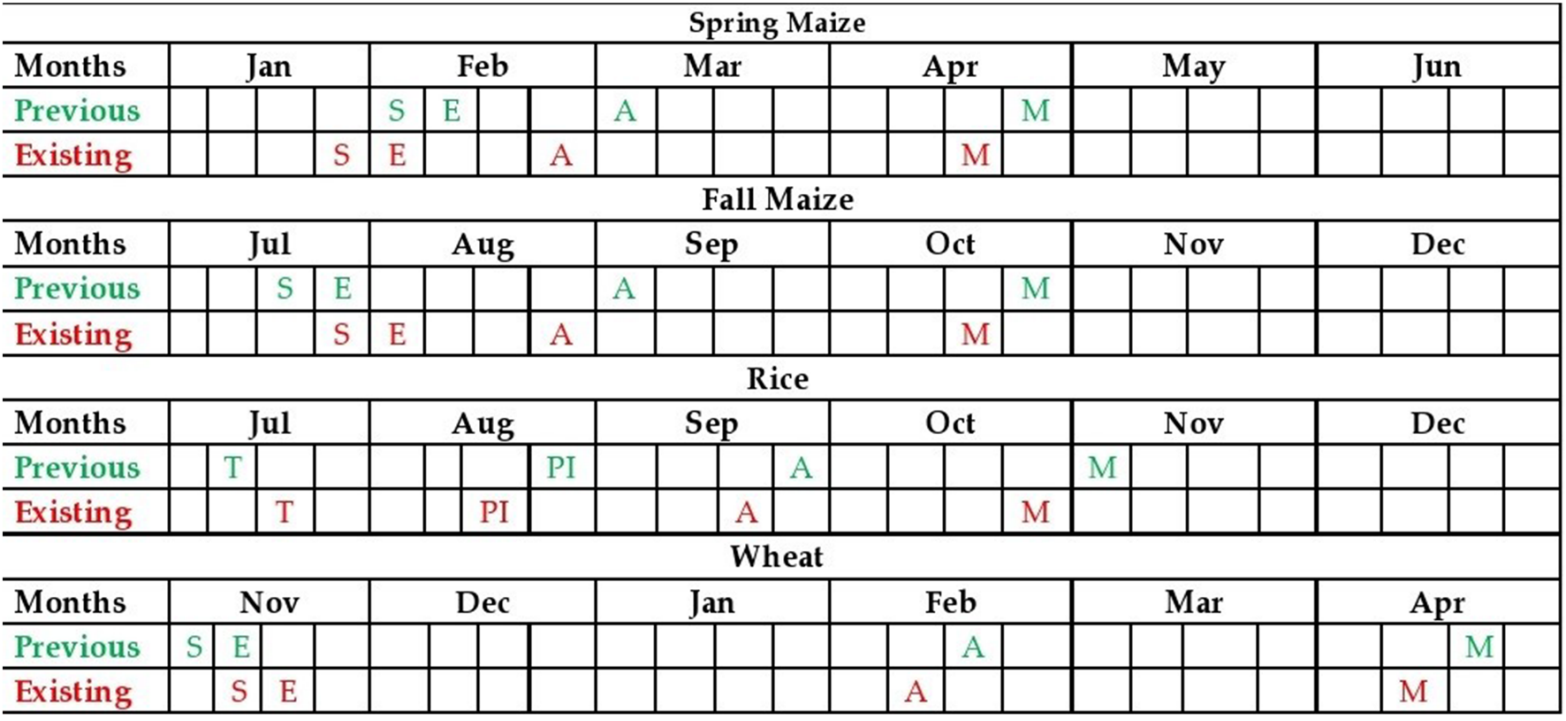
Spatial and temporal variability of spring maize, fall maize, rice and wheat crops phenological phases as effected by climate warming in Punjab, Pakistan. *S* Sowing, *E* Emergence, *A* Anthesis, *M* Maturity, *T* Transplanting, *PI* Panicle Initiation. Alphabets with green and red colors represent previous and prevailing trends. [Modified and adapted from Abbas et al.^109^ and Ahmad et al.^88^].

Phenological stages and phases of rice were affected by the worldwide thermal trend. Table 5 shows that sowing, transplanting (T), anthesis and maturity were earlier by 7.9, 6.6, 5.0 and 5.0 days decade^−1^, respectively in Punjab, Pakistan (Fig. 4) during 1980 to 2014^88^. Both the highest and lowest transplanting-to-maturity phases were reduced in Pakistan and China, respectively. Shrestha et al.^108^ reported that sowing, transplanting, anthesis and maturity were earlier by 5.4, 3.2, 6.2 and 4.8 days decade^−1^ in Madagascar. The phenological phases of sowing to transplanting, transplanting to anthesis and A-M were reduced by 2.9, 1.6 and 5.2 days decade^−1^, respectively in China during 1981 to 2009 as well as in other parts of world^46,70,104,120–122^. Transplanting to maturity was more severely reduced than other phenological phases (Table 5).

### Maize phenology trend

Phenological stages of maize were delayed in most countries (Table 5). Phenological phases of maize were also reduced in Pakistan (Fig. 4) during 1980 to 2014^109^. Lowest reduction of phenological phases was in China^102^. In USA, phenological stages S, E, A and M were earlier by 3.9, 3.2, 1.7 and 2.9 days decade^−1^, respectively, while, S-A, A-M and S-M were shortened by 2.9, 1.8 and 3.0 days decade^−1^, respectively during 1981 to 2005^123,124^. The S, E, A, and M were delayed by 3.0, 1.9, 2.8 and 4.4 days decade^−1^ in Pakistan during 1980 to 2014^109^. Phenological phases S-A, A-M and S-M were reduced by 3.1, 7.2 and 4.9 days decade^−1^, respectively in Germany during 1961–2000^8^.

### Oat phenology trend

Phenology of oat was affected due to climate warming. Table 5 shows that in Germany during 1959 to 2009, S, E, A and M of oat were earlier by 1.1, 1.8, 9.7 and 10.7 days decade^−1^ and phases S-A, A-M and S-M were significantly reduced by 7.9, 13.9 and 9.4 days decade^−1^, respectively. Estrella et al.^110^ reported that during 1951 to 2004, sowing, emergence, flowering and harvesting of oat were earlier by 1.5, 1.2, 4.9 and 6.4 days decade^−1^ and S-A, A-M besides S-M phases were significantly shortened by 3.4, 1.5 and 4.9 days decade^−1^, respectively.

### Barley phenology trend

Phenological stages of barley were earlier in Lithuania and delayed in Spain with global warming (Table 5). Sujetovienė et al.^43^ observed that S, E, A and M were delayed by 1.7, 2.8, 1.1 and 0.4 days decade^−1^ and phases S-A, A-M and S-M were reduced by 1.0, 1.6 and 2.2 days decade^−1^, respectively during 1961 to 2015 in Lithuania (Table 5). Phenological phases were reduced by 1.6, 2.1 and 3.7 days decade^−1^ for S-A, A-M and S-M, correspondingly in Spain during 1986 to 2008^81^.

### Rye phenology trend

Phenological phases of rye were also reduced in Germany and minimally in Poland^125^ (Table 5). The S, E, A and M were delayed by 2.2, 1.9, 4.0 and 3.6 days decade^−1^, respectively and phases S-A (1.8 days decade^−1^), A-M (1.4 days decade^−1^) and S-M (3.2 days decade^−1^) were reduced in Poland during 1957 to 2012. Phenological stages S, E, A and M were earlier by 1.0, 1.2, 1.8 and 1.6 days decade^−1^ and phases S-A, A-M and S-M were shortened by 2.9, 3.1, 4.5 days decade^−1^, respectively in Germany during 1960–2013 (Table 5).

## Effect of phenological shifts on crop yield

Climate warming is shifting the phenological stages of the crop. Crop yield is very sensitive to the accumulation of heat units or GDD during specific phenological events, thus by shifting the phenology, crop yield is also affected. Several studies reported/projected reduction in crop yields with climate warming due to shortening of phenological events without considering management practices^37,137,139,148^. The climate change adaptation strategies like cultivar shift and change of sowing dates could compensate warming effects. Although vegetative and reproductive phases are equally prone to climate warming^37,145,149^, the impact of high temperature on pollen viability, fertilization and post-fertilization processes leads to a marked decrease in final yield^16,145,150^. However, all phenological stages are not equally responsive to climate warming effects and do not have equal impacts to final yield^12,103,127,151–153^. Tao et al.^38^ investigated the effects of phenology shift on yield of wheat, maize and rice during 1981 to 2000. They reported that earlier planting of 3–6 days decade^−1^ and earlier anthesis dates decreased yield up to 110 kg/ha/yr in wheat, 168 kg/ha/yr in maize, and 20 kg/ha/yr in rice. Zacharias et al.^154^ investigated the effect of induced high temperature on phenology and yield of wheat and rice cultivars. They observed that high temperature advanced dates of anthesis and crop maturity, and reduced the duration of vegetative and maturity phases, and led to reduction in yield. They reported 26% reduction in grain yield of rice due to reduction in the anthesis and maturity dates of around seven to 8 days, respectively. The reduction in yield was primarily attributed to less time for photosynthesis and accumulation of assimilates. Warmer temperature during the reproductive phase led to pollen sterility and high evapotranspiration, which led to fewer grains and lower grain weight, reduced photosynthetic rate, and reduced duration of phenological events^90,91^. Hatfield and Prueger^90^ reported that temperature extremes during the reproductive phase could reduce the grain yield of maize up to 90% mainly due to pollen sterility and smaller grain size. Nahar et al.^150^ investigated the impact of climate warming on five cultivars of wheat. In their studies heat stress imposed by late sowing of the crop resulted in significant reduction in days to booting, anthesis and maturity across all cultivars (although cultivar differences existed). About 8–13 days reduction in days to anthesis and maturity resulted in 53–73% yield losses in different cultivars. Meanwhile, Sadok and Jagadish^155^ reported that nighttime warming poses a threat to global food security (6% decrease in winter wheat yield per 1 °C rise in night temperature while 4–7% reduction in spring wheat and rice yield per 1 °C rise in night temperature) as it is driving yield declines worldwide. Thus, they proposed ecophysiological framework as a guide to implement future research efforts to mitigate yield declines. They further elaborated that efforts should include integrated approaches i.e. physiology with crop modeling, breeding and management to intensify sustainable pathways for mitigation as intensity of climate change is becoming stronger and stronger day by day.

Climate warming significantly impacts the crop phenology, which in turn leads to a reduction in yield mainly due to shortened phenological events coupled with less production of assimilates, pollen sterility and biochemical irregularities^156^. The climate warming impacts have been neutralized to some extend with shifting of sowing date and development of cultivars with longer duration of phenological events even under warming conditions. As climate warming is a continuous process, therefore, integrated research is required to understand the mitigation of climate warming impact for sustainable crop production to feed an ever-increasing world population.

## Consequences of climate warming

**Figure 5:**
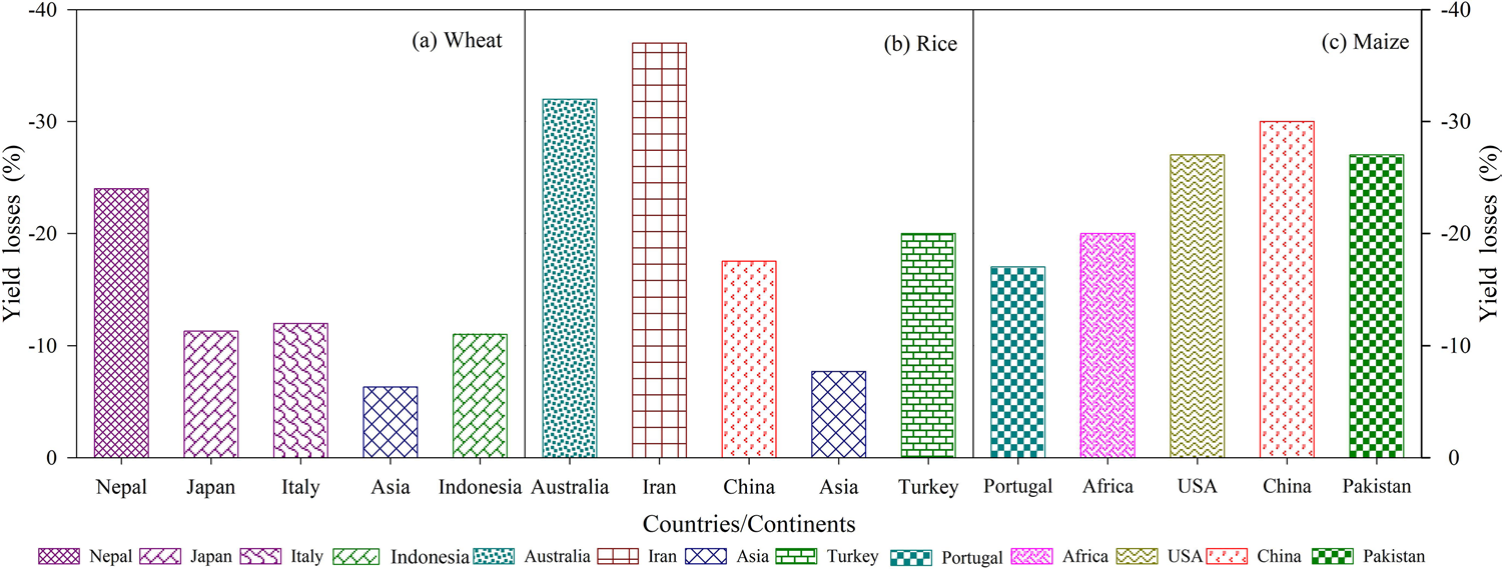
Effect of climate warming on productivity of primary cereal crops: wheat, rice and maize across various countries/continents. [Modified and adapted from Ishfaq et al.^192^].

Climate warming is threatening all living organisms including human beings. Rising temperatures at the poles and glaciers, increase the risk of floods, and many populated areas may be inundated, and fertile lands may become deserts^157–161^. The main consequences linked with a rise in sea level and desertification are salinity, heat and drought stresses, and these factors are major threats to agricultural production including primary cereals (Fig. 5).

## Adaptation strategies in response to climate change

Adaptation strategies in CSA are the only way to reduce negative effects of climate warming on cereal crops. Different adaptation approaches like better crop management practices, modern breeding, and biotechnological approaches can develop climate resilient cereals to help cope with climate warming^56,105,106,111,127,162–165^. Revolutions in plant breeding (molecular and conventional for incorporation desired characteristics) and genetic engineering (integrated transgenic techniques like marker-trait associations and genome-wide selection) could assist to reduce overwhelming food security concerns against extreme-weather conditions, through producing climate adaptive cereal plants^166–173^. Thus coping with climate warming is an urgent concern globally and in order to adapt cereals to changing climatic conditions, the following approaches are mandatory.

### Crop management practices

Better and improved crop management practices can minimize negative impacts of temperature extremes. Under climate warming, all better crop management practices should be brought to the fields to get real benefits^105,129,174–186^. Usage of quality seed are big concerns and quality seed availability to the farmers are under 20%. Good quality and genetically pure seed should be provided to farmers to minimize negative impacts of climate change and achieve sustainable nutrition security^6^. Farmers’ access to good quality seed of cultivars adapted to diverse ranges is still obstructed by inadequate and incompetent seed production besides weak delivery systems, scanty seed policies, and excessive price of seed^187^. There are many helpful adaptation strategies related to crop husbandry reported by scientists, comprising abiotic factors, for example, changing sowing and harvesting date, crop rotation, irrigation techniques, deficit irrigation, soil management practices, improvement of irrigation efficiency and variation in cropping patterns etc.^5,14,25,188–191^ (Table 6).

**Table 6:** Observed changes in trend of phenology of agronomic crops in different countries.

Crop	Continent	Adaptation strategies	References
Wheat	Asia, Spain, Australia, South America, Europe	Early Sowing, Sowing/planting dates adjustment	^5,25,60,81,88,101,109,140,189,192–200^
	Asia	Cultivars requiring higher GDDs; Improved cultivars; Use of heat tolerant cultivars;	^14,109,127,140,198,201^
	Asia, South America	Optimum plant population	^178,198^
Rice	Asia	Sowing date	^88,144,202,203^
	Asia	Varieties requiring higher GDDs	^70,106,107,204–208^
	Asia	Direct planting	^209,210^
	Asia	Early maturing, Climate ready rice	^211,212^
	Asia	System of rice intensification with alternate wetting and drying	^213^
	Asia	Improved variety and management	^214^
Maize	South Asia	Sowing date	^78,92,140,141,145,149,215^
	Asia, America	Early maturing cultivars; Varieties requiring higher GDDs	^144,216^
	Asia	Raised bed planting	^216–220^
	Asia	Precision nutrient management	^221^
Oat	Europe	Varieties with larger thermal time	^52^
Rye	Poland, Europe	Late maturing cultivars, Plant density	^60,125,134,199,200^
Millet	South Asia	Planting time; Plant spacing	^222–224^

Raised bed planting method for maize and rye crop is useful to obtain maximum attainable grain yield. The most important approach is choosing the optimum sowing date for cereal crops under climate change. Changing sowing date to evade the harmful influences of high temperature at anthesis and during pollination and fertilization has been suggested as an adaptation tactic for climate warming. A change in sowing window, for example, earlier sowing of winter and spring cereal crops and delayed sowing of summer and autumn cereals to escape hot and dry periods in growing seasons, is one useful adaptation to climate uncertainty. Therefore, sowing of cereals has been adjusted according to optimum temperature conditions, resulting in substantial yield increases by escaping temperature stress during grain filling phase^60,127^. Optimization of sowing time, planting density, and best irrigation techniques are dynamic strategies to confront weather-based stresses. Establishing innovative and precision irrigation water management might contribute to the mitigation of negative issues related to climate change. Better irrigation management can improve cereal crop productivity under climate warming. Water-saving interventions such as direct-planted rice, a modified system of rice intensification and alternate wetting and drying (AWD) of rice were implemented in a cluster approach and resulted in enhanced water use efficiency^225–229^. Optimum fertilization rates are also very effective to decrease the impact of global warming. Optimizing fertilizer use provides for better adaptability by enhancing energy capture by plants, by maintaining soil fertility and pesticide use, and increasing productivity^230–233^. The significance of fertilizer in nourishing the world population is undeniable. Enhancing fertilizer use efficiency (minimizing losses like volatilization and leaching; ultimately nutrients availability to crops) is dynamic under climate change. Adaptation of integrated soil fertility management and precision nutrient management is helpful to minimize the negative influence of higher temperature^221,234,235^. Laser land leveling (through enhancing water application efficiency, uniform distribution in fields and availability to crop plants; thus increasing water use efficiency) can enhance cereal grain yield under warming climate. Agroforestry techniques could help growers to diversify their crops, and their profits, along with enhancing climate resilience. Integrated pest management are useful approaches to cope with the thermal trend impact in cereal based cropping systems^173,235,236^. Some important agronomic practices to cope with climate warming are described below in greater detail.

### Sowing date

Adjustment of sowing date to accommodate the thermal trend is significant for proper phenology of cereal crops. Zhang et al.^237^ reported that delayed sowing might be an efficient way to coup with water stress. Climate warming accelerates crop growth and shortens duration of phenological phases of cereals. Physiological processes including photosynthesis, starch conversion and nutrient metabolism are negatively affected by changing phenology due to heat stress^31,200,238–241^. The use of climate-smart cultivars, besides optimum planting dates, can reduce risk impacts due to warming^149,242^. Hence, decisions about seed rate, planting density, spatial arrangement and other management practices including irrigation, nutrition and application of pesticides etc. are affected by planting date^243–245^. However, to get real benefits sowing date should coincide with growth cycle to make the optimal use of possible environmental circumstances and managements^149,202,203,246–248^.

### Planting geometry

Optimum planting geometry can be employed for the reduction of harmful effects on phenology due to climate change. Plant phenology is also affected by planting geometry^130,249^. Crop microclimate is changed by varying planting geometry like planting density, row spacing, seed rate etc. Management of planting geometry, e.g., decreased plant density, broader plant or row to row distance, and skip-row configurations are adaptation strategies that can be adapted in the dryland farming areas for better utilization of available soil-water^250^. Nevertheless, optimum planting geometry could improve resource use efficiencies in crops through proper crop phenology, producing more leaf area and ultimately more favorable yield components and yield in cereal crops in all climatic conditions^251,252^. Planting geometry affects solar radiation interception, canopy coverage, crop growth rate and biomass accumulation^215,253–258^. Furthermore, with optimum planting geometry, total dry matter production and ultimately grain yield increase was mainly due to more photo-assimilates^252^. Planting practices have significant impact on maize productivity as concluded by Huang et al.^259^. They reported that soil temperature and growth period precipitation are determinant factors in dryland farming systems. Thus, they recommended that selection of suitable planting patterns according to the limiting factors can uplift dryland agriculture.

### Supplementary irrigations

Supplemental irrigation is an important adaptation strategy for the reduction of harmful effects due to climate warming on cereal crops worldwide^260^. Supplemental irrigation is application of a limited amount of water at very critical stages for the improvement and stabilization of grain yield of cereal crops when rainfall is insufficient to provide adequate water for proper growth and development. Supplemental irrigation can minimize the impact of heat stress^261^. During the previous decades, the main source of water was canal irrigation water, but nowadays, water shortage is severe, so supplemental irrigation can be helpful especially in arid environments. Supplementary irrigation, particularly during critical crop phenological stages and phases, can improve cereal crop yield as well as water efficiency in cereal based cropping systems^262^. Supplementary irrigation practice is a simple, nevertheless, extremely effective practice that allows the farming community to grow and manage cereal crops by irrigating at the optimum time, without being at mercy of the unpredictable precipitation. Supplementary irrigation permits farmers to grow cereal crops during their optimum growing period, which can enhance grain yield and avoid crop exposure to lethal heat and drought stresses in warm areas, and frost in cooler areas worldwide^263^.

### Plant breeding and genetic modifications

Plant breeding and genetics provide dynamic mechanisms for adaptation of cereal cropping systems to heat stress. The combination of molecular and conventional plant breeding and genetic methods can be helpful to recognize and develop eco-stable varieties with required genotype-environment combinations that will be beneficial in farming under changing climatic circumstances^166,167,264–267^. The desirable characters like drought, and cold and heat stress resistance, resistance to pests, and ability to cope with water logging and salinity can be accomplished by integrating conventional, molecular and transgenic techniques^169,170,268–270^. Zachariah et al.^271^ recommended use of drought-resilient food crops to mitigate the immediate agrarian crisis. As according to their analysis, rainfall deficit is the primary cause of crop failure, as compared to rising temperatures. Comprehensive overview of the C5-MTase gene family members in wheat was presented by Gahlaut et al.^272^. They identified 52 C5-MTases (cytosine-5 DNA methyltransferases) gene family members (4 sub-families i.e. CMTs (Chromomethylase), METs (Methyltransferase), DRMs (Domains Rearranged Methyltransferase) and DNMT2s (DNA methyltransferase homologue 2)) that could be used to develop drought/heat stress in wheat.

Molecular approaches, comprising the use of marker-trait associations and genome-wide selection, are providing opportunities for developing germplasm with tolerance to several abiotic and biotic stresses. The adverse effects of heat stress can be mitigated by developing crops with enhanced thermo-tolerance. Boote et al.^273^ in their work concluded that peanut, soybean, pearl millet are more heat tolerant than sorghum, bean and chickpea. Genetic diversity for climate change has previously been reported for cereals with the application of comparative biology, genetic diversity analysis, collaborative phenotyping and crop simulation modeling^198,274,275^. Photoperiod insensitivity could be incorporated by breeders; accordingly seed-filling could start before onset of higher temperatures. The use of modern cereal crop varieties is dynamic adaptation strategy for obtaining higher grain yields under climate change^274^. Adoption of improved cultivars is an important approach to adapt to climate variability and change. Heat tolerant varieties for cereal crops are essential under higher temperature stress^198^. Breeding and sowing of improved, resistant cultivars (against abiotic and biotic) are key strategies to adapt to climate change. Growing varieties with enhanced climate warming tolerance is important to maintain pollen viability, for example, grain number and grain protein^163,275,276^. Late-maturing maize cultivars enhance grain yield due to longer grain filling duration. Improved cultivars with resistances and tolerances to abiotic and biotic stresses for cereal crops can increase resource use efficiency. Growing of genotypes requiring more GDDs can better resist thermal stress (Table 6). Drought tolerant varieties can increase radiation use efficiency under climate warming conditions. Avoiding drought stress by genetic improvements will have a major role for ensuring food security and reducing grower's exposure to drought-risk. Adapting cultivars with deep root-systems, mainly in areas that experience prolonged dry spells, could be a useful strategy under climate warming stress. Slow maturing cultivars also minimize the adverse effects of heat stress particularly at anthesis^144^.

### Biodiversity

Agricultural biodiversity is essential for adapting to climate change (i.e., multiple cropping vs. sole cropping; diversified/integrated farming vs. specialized farming). Growing different varieties of crop can be beneficial in tackling climate-warming risk^277–279^. More diverse and longer-cycle crop rotations will need to combine sequences of annual row crops such as maize and soybean with close-drilled cereals, shallow-rooted with deep-rooted crops, summer crops with winter crops, and annuals with perennials in the same fields^280–283^. Similarly, increasing diversity within crops may be a powerful way to reduce agricultural declines from climate change as concluded by Morales-Castilla et al.^284^. Multi cropping systems and crop rotation (including leguminous or green manuring crops in existing cereal-based cropping systems) are useful to combat the adverse influence of climate change. Resilience to unpredictable weather will also benefit from intercropping, with the creative arrangement of multiple interacting crop species to diversify the field and the landscape^101,172,285,286^. Sloat et al.^287^ reported that warming impact on maize, wheat and rice could be moderated by migration of these crops over time and the expansion of irrigation. Multiple-cropping systems and strategies to integrate animals and crops will make more efficient use of natural resources and applied inputs; these include systems such as permaculture, agroforestry, alley cropping, intercropping and sowing C_4_ crops compared to C_3_ crops. Diverse cropping systems with spatial diversity, and adapted to specific fields, soil conditions, and unique agro-ecozones can minimize negative effects of heat stress^288^. Cereal crop production resilience can be achieved by planting diverse combinations of cropping schemes together in the same field, and economic resilience through production of a range of products that can be marketed by various channels^215,253,289,290^. Multiple cropping systems can diversify the cropping systems. This comprised of sowing two or more cereal crops on the same field, either at the same time or one after another, are production intensification strategies^215,253^. Such cropping systems have the benefits of decreasing the risk of whole crop failures, therefore ensuring a higher level of production stability for the farm community^235,291^. Sufficient cropping systems, particularly in regions that are extremely influenced by the effects of climate warming, are professed as an important approach to adaptation. Grain yields in sequential cropping systems were higher than average grain yields in single cropping systems, suggesting that sequential cropping systems contributed towards reducing the negative impact of climate warming in comparison to single cropping systems^83,292–294^. Resource use efficiency through crop diversification can further be improved by adapting advanced production technologies, physiological based resilience to climate change, crop rotations, improved water and nitrogen use efficiencies, crop modeling as well as planning before time through the use of forecasting skills to adapt to climate change under current and future climate scenarios^163,196,286,290,295–314^.

## Conclusion

This work has highlighted the negative impacts of climate warming on cereals crop phenology which will become frequent and severe in future. Climate warming is threatening global food security due to consequences like heat, drought, and salinity stresses. It has shown considerable impact on cereal production by shortening crop phenology. With climate warming, all phenological events occurred earlier and resulting phenological phases were shortened in various regions and countries across the globe, as a result of which plants got less time to assimilate CO_2,_ and this ultimately resulted in yield reduction. The earlier wheat stages were observed in northwest China, and delaying was recorded in north south regions in China and Pakistan. The reduction in phenological phases was also observed in northeast China, Pakistan, Spain, Australia, Argentina, Romania, Germany and Pakistan. Rice phenological stages and phases were affected worldwide. The rice stages were earlier in Punjab, Pakistan and India, and transplanting-to-maturity phases were reduced in Pakistan and China. Maize phenological stages were delayed in most countries. Phenological phases of maize were also shortened in Pakistan, China and USA. In Germany and in Lithuania, oat stages were earlier while delayed in Spain due to global warming. However, these phases were significantly reduced in Spain. Rye phenological stages were earlier, and phases were shortened in Poland and Germany.

## Recommendations

This impact of climate warming can be mitigated by developing cereal crop cultivars well adapted to climate change, changes in management practices e.g. sowing dates zoning of crops and by increasing cereal crop biodiversity. Further research regarding crop plasticity is necessary to adapt cereal crops under a wide range of climatic conditions in order to avoid yield losses due to climate warming. Similarly, wider spectrum climate change adaptation options e.g. change in cropping patterns, use of early maturity and less water-consuming crop varieties, availability of stress tolerant crop varieties, modification in sowing date, crop insurance, implementation of location specific technologies, application of modern technology, intercropping, mixed cropping, socioeconomic and institutional interventions, crop modeling, whole farm modeling, integrated crop-livestock management and plantation of tress around the field are recommended to mitigate the impact of climate warming.
